# Early-Stage Melanoma Benchmark Dataset

**DOI:** 10.3390/cancers17152476

**Published:** 2025-07-26

**Authors:** Aleksandra Dzieniszewska, Piotr Garbat, Paweł Pietkiewicz, Ryszard Piramidowicz

**Affiliations:** 1 Institute of Microelectronics and Optoelectronics, Warsaw University of Technology, 00-661 Warsaw, Poland; piotr.garbat@pw.edu.pl (P.G.); ryszard.piramidowicz@pw.edu.pl (R.P.); 2 Unitelematica Leonardo da Vinci, 6300 Zug, Switzerland; pietkiewicz.pp@gmail.com; 3 Centrum Medyczne Zwierzyniecka, 60-465 Poznań, Poland

**Keywords:** skin lesion diagnosis, melanoma, benchmark, deep-learning, T-category, Breslow thickness

## Abstract

Background: The early detection of melanoma is crucial for improving patient outcomes, as survival rates decline dramatically with disease progression. Despite significant achievements in deep learning methods for skin lesion analysis, several challenges limit their effectiveness in clinical practice. One of the key issues is the lack of knowledge about the melanoma stage distribution in the training data, raising concerns about the ability of these models to detect early-stage melanoma accurately. Additionally, publicly available datasets that include detailed information on melanoma stage and tumor thickness remain scarce, restricting researchers from developing and benchmarking methods specifically tailored for early diagnosis. Another major limitation is the lack of cross-dataset evaluations. Most deep learning models are tested on the same dataset they were trained on, so they fail to assess their generalization ability when applied to unseen data. This reduces their reliability in real-world clinical settings. Methods: We introduce an early-stage melanoma benchmark dataset to address these issues, featuring images labeled according to T-category based on Breslow thickness. Results: We evaluated several state-of-the-art deep learning models on this dataset and observed a significant drop in performance compared to their results on the ISIC Challenge datasets. Conclusions: This finding highlights the models’ limited capability in detecting early-stage melanoma. This work seeks to advance the development and clinical applicability of automated melanoma diagnostic systems by providing a resource for T-category-specific analysis and supporting cross-dataset evaluation.

## Simple Summary

The Early-Stage Melanoma Benchmark (EMB) dataset was designed to support the development and evaluation of deep learning models for early melanoma detection. Existing datasets often lack information on melanoma stage or Breslow thickness, limiting researchers’ ability to accurately assess models in the early-stage melanoma detection task. EMB addresses this gap by providing over 1100 dermoscopic and clinical melanoma images labeled according to T-category in TNM classification. The dataset is curated from public sources and filtered to avoid overlap with ISIC training data. Several state-of-the-art models were evaluated on EMB, revealing a significant performance drop, particularly for in situ and thin melanomas, compared to results on standard ISIC datasets. This highlights the challenges of early-stage detection and the need for dedicated datasets. EMB enables cross-dataset benchmarking and T-category-specific analysis, offering a valuable resource for assessing clinical applicability and the robustness of automated melanoma diagnosis systems.

## 1. Introduction

Melanoma is one of the deadliest forms of skin cancer, but early detection significantly improves the chances of successful treatment. According to the U.S. National Cancer Institute, the 5-year survival rate for patients diagnosed at localized stages (0, I, II) is as high as 97.6%. In contrast, survival rates decrease sharply to 60.3% when diagnosed at a regional stage (III) and drop further to 16.2% at the metastatic stage (IV) [1]. These statistics highlight the importance of melanoma diagnosis in the early stages.

Recent advances in deep learning have accelerated the development of automated methods for skin lesion diagnosis, leading to a growing number of AI models designed specifically for dermatology [2,3,4,5]. The availability of relatively large, publicly accessible skin lesion datasets has further propelled progress in this field, enabling the refinement of deep-learning-based diagnostic approaches. The adoption of such automated systems for melanoma detection holds significant potential for improving early diagnosis and, consequently, enhancing patient survival rates.

Numerous studies have explored early melanoma detection using dermoscopic images with deep learning techniques [6]. For instance, Alenezi et al. [7] used a lesion segmentation mask to extract the area of interest for subsequent classification to improve melanoma diagnosis. Lin et al. [4] used YOLOv5 for the detection of different melanoma types on RGB images converted into hyperspectral images. A framework for explainable melanoma diagnosis that integrates features extracted from the lesion, population information, and patient metadata was developed by Akash et al. [8]. This framework uses a transformer architecture that allows for the leveraging of evidence based on global context derived from all patient lesions and associated metadata. Other notable contributions were made by Elshahawy et al. [5], who used YOLOv5 for melanoma detection on dermoscopic images in real time. Mahmud et al. [9] proposed an Xception-based method for early-stage melanoma detection. They also used extensive preprocessing techniques, including artifact removal and hair removal, along with data augmentation, to improve model generalization. While these described methods focus on early-stage melanoma detection, a critical gap persists, i.e., they frequently lack an analysis of model performance that explicitly verifies their efficiency on early-stage melanomas, often relying on broader performance metrics that may not fully capture this specific capability. Yu et al. [10] addressed the important issue of neglecting temporal dependencies in melanoma progression. Their approach leverages changes in the lesion over time to enhance early melanoma detection. This is accomplished by aggregating information from temporally aligned sequential images through a spatio-temporal network. Additionally, they implemented knowledge distillation to transfer insights from later time points to earlier ones by penalizing inconsistent predictions across different time steps.

Several dermatological AI methods have also been proposed for classifying Breslow thickness into intervals. For example, Polesie et al. [11] show that clinicians outperform a de novo convolutional neural network in classification between in situ and invasive melanoma, but pre-trained ResNet-50 achieved similar performance to the human readers. It is a challenging task due to the limited availability of data. To mitigate the scarcity of Breslow thickness-labeled images, knowledge distillation was employed to enhance classification performance in two tasks: distinguishing in situ from invasive melanoma, and categorizing melanoma into in situ, thin, and thick classes [12]. A different approach was proposed by Jaworek-Korjakowska et al. [13], who used the SMOTE oversampling method and the VGG-19 model to classify Breslow thickness into three intervals. Nogales et al. [14] used a pretrained ConvNeXt and focal loss for the classification of melanoma into two classes based on Breslow depth. Described studies rely either on private or difficult-to-access datasets [11,13] or on Breslow thickness labels that do not correspond to the T-category definitions of the AJCC staging system [15].

Despite recent progress, existing lesion analysis methods face several limitations. First, the majority of datasets suffer from a lack of detailed metadata from pathology reports such as Breslow thickness, presence of ulceration, regression, dermal mitotic rate, tumor-infiltrating lymphocytes, preexisting nevus, satelitosis, and perivascular and perineural invasion, making it difficult to assess how well these methods perform, especially on early-stage lesions. Specifically, Breslow thickness and ulceration determine the T-category in the TNM classification used for disease staging. Without this information, the distribution of early/advanced melanomas remains unknown. Since early detection of melanoma is a primary objective of deep learning models, the lack of dedicated datasets makes it difficult to assess their effectiveness accurately. This gap poses a significant challenge to the development of reliable diagnostic tools, as early-stage melanoma can exhibit visual similarities to benign nevi, borderline lesions, melanosis, or even scars [16]. These variations in appearance across stages could pose a challenge for deep learning models, potentially affecting classification performance. Additionally, early-stage melanoma often exhibits subtle features, making it more challenging to differentiate from benign lesions and complicating accurate diagnosis. As a result, an imbalanced distribution of melanoma stages in datasets can introduce bias into deep learning models. When trained on data with unknown stage distribution, models may not effectively address early detection, presumably failing to identify early-stage melanoma accurately.

Secondly, the wide availability of large open-access datasets presents a challenge, as nearly all research projects rely on the same training data, while cross-dataset evaluation remains vastly underutilized. For example, only 36 of 176 skin lesion segmentation methods studied in [17] used cross-dataset evaluation to test their models. Moreover, only 23.6% of works studied in [18] used an external dataset for evaluation. Without testing across diverse datasets, it is challenging to determine whether a method will perform robustly in real-world clinical settings.

Assessing the performance of deep learning models is a critical medical and legal concern, prompting the curation of specialized datasets for this purpose. Researchers can use general benchmarks to evaluate the robustness of deep learning models [19]. Additionally, specialized skin lesion analysis datasets exist, such as skin tone diversity benchmarks for lesion diagnosis [20,21] and datasets that categorize images by Breslow thickness bins rather than providing exact values [15]. While there are multiple datasets containing skin lesion images, it is not a common practice that models are tested with multiple datasets from different sources, and no further analysis of the bias and robustness of the models is provided [18].

We propose an early-stage melanoma benchmark dataset (EMB) consisting of melanoma images with Breslow thickness and T-category. Data was collected from known public sources and delivered in a standardized format for future use. We also present a detailed evaluation of multiple models on the curated dataset. Our results confirm that models perform worse on a dataset composed of thin melanoma images compared to on a dataset with an unknown stage distribution. The EMB dataset addresses the gaps described above as it provides labeled images with an emphasis on early-stage melanoma and is designed to facilitate cross-dataset benchmarking, offering a new standard for assessing the reliability and accuracy of automated melanoma diagnostic tools. The dataset and code used to prepare the data are available at GitHub https://github.com/Oichii/EMB (accessed on 20 June 2025).

## 2. Methods

The early-stage melanoma benchmark dataset was developed to support research on automated melanoma diagnosis with a particular focus on early detection. It consists of melanoma images paired with corresponding thickness information provided in millimeters. The thickness data was extracted from image descriptions or metadata found in archival sources. Each lesion was categorized into a T-category label based on its thickness.

### 2.1. Data Collection

Images for the datasets came from two publicly available repositories: the ISIC Archive [22] and the Dermoscopy Atlas website [23]. From the ISIC Archive, images with a melanoma thickness label and images from the category melanoma in situ were downloaded with corresponding metadata using the provided interface. The rest of the data was scraped from the Dermoscopy Atlas website. For scraped images, the thickness label was extracted from the image description or the assigned diagnosis of melanoma in situ. Initially, we collected 1290 images with matching Breslow thickness.

### 2.2. T-Category Label

Online image repositories are based on community contribution, which causes the issue of potentially duplicated images appearing in the same repository and the same image being present in multiple repositories. Possible overlap between our data and the datasets commonly used for training was also assessed. Most skin lesion diagnosis methods were trained on ISIC datasets from the years 2016–2020 [24,25,26,27,28,29], so we removed overlapping images between our benchmark dataset and ISIC challenge datasets to ensure a fair comparison.

Following [30], we found duplicates using the *czkawka* [31] application that compares image hashes using hamming distance to find identical and similar images. The search was performed using the Lanczos3 resize algorithm and gradient type hashes of size 16. The similarity threshold was set to 40 to find images with even minimal resemblance. All flagged duplicates were manually investigated to exclude false positives. We do not consider multiple images of the same lesion as duplicates. We began by identifying duplicate images within the collected dataset, where we found and removed 7 identical images. Next, we searched for duplicates between the ISIC Challenges and our benchmark datasets. Initially, this search was based on ISIC IDs, which led to us identifying and removing 146 duplicate images. Additionally, using the *czkawka* tool, we detected 33 more duplicate images with different ISIC IDs.

Currently, melanoma stages are defined based on metrics proposed by the American Joint Committee on Cancer (AJCC) using, among others, lesion thicknesses [32]. We assigned the labels based on the T-grouping of the AJCC staging method. Breslow thickness indicates tumor thickness from the granular layer of the epidermis to the deepest level of invasion [32]. We assigned T-category labels based on thickness information according to the Table 1. We grouped the a and b categories together as we did not have ulceration information to distinguish them.

**Figure 1:**
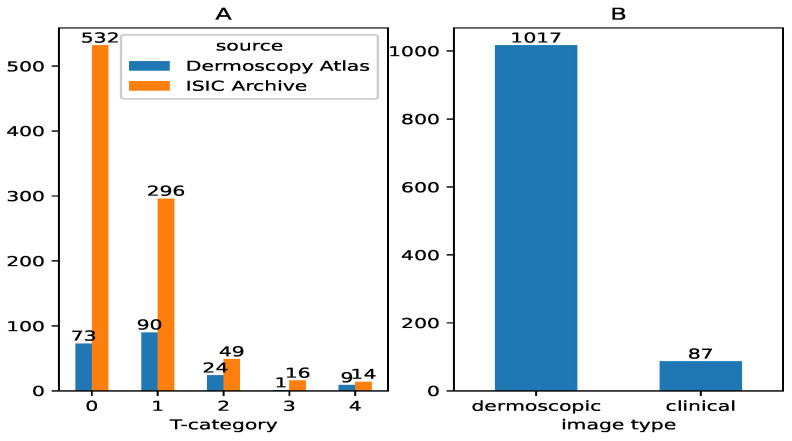
Composition of benchmark dataset (**A**) by source and T-category of melanoma, and (**B**) by image type (dermoscopic vs. clinical).

Figure 1 presents the final composition of the dataset. A total of 1104 images with thickness information were collected. Of these, 907 images came from the ISIC archive, and 197 were obtained through web scraping from the Dermoscopy Atlas website. The dataset includes 1017 dermoscopic images and 87 clinical photographs. Atlas data did not include the image type, so we labeled it based on visual characteristics observed in the data.

## 3. Results

To establish benchmark results on the EMB dataset, we tested various methods with code and model weights available online. We tested EfficientNet Ensemble, the winner of the SIIM-ISIC challenge in 2020 [33]; Class-Enhancement Contrastive Learning for Long-tailed Skin Lesion Classification (ECL) [34]; and deep mask pixel-wise supervision (DMP) [35]. All methods were trained on data from the ISIC challenge datasets without external data. These methods were selected based on the public availability of the code; trained models; and the demonstration of SOTA performance. Clinical images were excluded from testing as the methods were trained on dermoscopic images only. For the ISIC 2020 winner, we tested individual models from the ensemble, selecting only those that did not utilize metadata. For ECL, we tested two versions of the model: the 8-class model and the 9-class model. Similarly, for deep pixel-wise supervision, we tested the ConvNeXt 9-class models and the 2-class models. Input images for each model were preprocessed according to the specific requirements described in the original papers. Classification accuracy was assessed by checking whether the model correctly predicted the melanoma/malignant class. Since our dataset contains only melanoma cases, all images should be classified as either the positive class in binary models or the melanoma category in multi-class models.

Results achieved by tested models are shown in Table 2. Column *All* shows results for the entire dataset, and Columns *Tis-T4* provide results for lesions in the corresponding thickness stages. Column *Rep.* shows the average accuracy reported by the authors on the ISIC dataset on which the model was tested.

All tested algorithms performed well on the datasets they were originally trained and evaluated on, but exhibited a significant drop in performance when tested on our dataset. The best-performing model from the ISIC winner ensemble (EfficientNet-B4 768 9c) reached an accuracy of 97.71% on the combined data from ISIC 2018-2020. However, when evaluated on our data, it achieved a median accuracy of 0.54 with a 95% confidence interval of [0.52, 0.57]. Similarly, the ECL 9 class model, which achieved an average accuracy of 86.11% on the ISIC 2019 test, showed a reduced accuracy of 0.40 (confidence interval [0.37, 0.42]) on EMB data. The deep mask pixel-wise supervision (ConvNeXt-DMP 224 2c) achieved 90.70% accuracy on ISIC 2019+2020 data, and on EMB, its accuracy declined to 0.55 with a confidence interval of [0.52, 0.58]. All models showed the lowest accuracy on in situ and T1 melanomas, indicating difficulty in detecting early-stage lesions. Performance generally improved with increasing thickness, with T3 and T4 melanomas yielding the highest accuracy for most models, although some achieved their best results on T2 cases.

**Figure 2:**
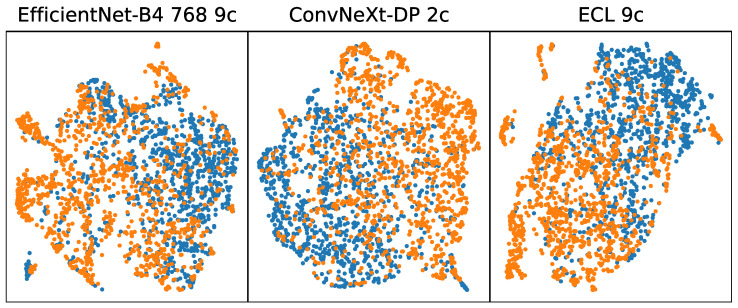
UMAP visualizations of features extracted by the final convolutional layer for selected models. Each point corresponds to a feature vector derived from one image, with blue indicating melanoma images from the ISIC 2019 Challenge dataset and orange indicating images from EMB. The figure illustrates differences in how each model clusters features, with non-uniformity visible for all three models.

Figure 2 shows a Uniform Manifold Approximation and Projection (UMAP) visualization of features extracted from melanoma images from the ISIC 2019 dataset (blue) and from EMB (orange). UMAP allows for the visualization of high-dimensional features on a 2D plane, which allows for the observation of the distribution and relationships between data points. For the well-trained model, one would expect a homogeneous and compact feature representation. In the case of the presented models, the visualization reveals a relatively uniform structure in feature representation for both datasets, yet non-uniformity exists between the challenge data and the benchmark data. This indicates that while early-stage melanoma shares some common features with later-stage melanoma, these shared features are insufficient for accurate classification when using models trained on the ISIC Challenge datasets.

**Figure 3:**
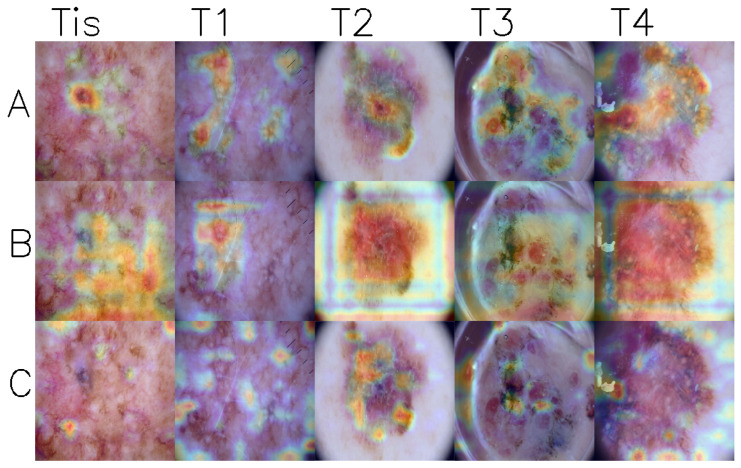
Grad-CAM visualization of gradients produced by the final convolutional layer of selected models. Red indicates regions that the model considers more important, and blue indicates areas of lower importance. Each column shows melanoma images in different stages (Tis–T4). Each row represents different model: (**A**)—EfficientNet-B4 768 9c, (**B**)—ConvNeXt-DP 2c, and (**C**)—ECL 9c.

Figure 3 shows Grad-CAM [36] visualizations of activation maps from the last convolutional layer for three models. Grad-CAM visualizations offer insights into the decision-making processes of the tested deep learning models by showing regions that are important for the prediction. Upon examining the images, it can be observed that models generally focus on the lesion areas, particularly in stages T2 to T4. However, in stages Tis and T1 cases, the models often concentrate on seemingly unrelated regions while ignoring areas with high pigmentation.

## 4. Limitations and Future Work

While our dataset serves as a benchmark for early melanoma diagnosis, several limitations should be considered. Although efforts were made to separate the benchmark dataset from the ISIC Challenge sets, some overlap may remain. Certain images in the benchmark dataset may appear in training sets if they originate from the ISIC Archive. This could affect benchmarking reliability, as models might previously be exposed to similar data in the training and validation stages. In addition, the dataset contains a higher representation of thin and in situ melanoma cases compared to more advanced stages. There is also a larger proportion of dermoscopic images relative to clinical images. The dataset prioritizes early intervention research, so we did not concentrate on balancing the dataset but rather on providing the highest possible number of melanoma images. Also, the dataset’s composition is derived from public repositories, inherently reflecting the “as-is” nature of available data, and thus was not specifically curated to mitigate a racial bias.

Future work will focus on developing a specialized model for melanoma T-category classification. This model will label data with an unknown T-category, which will then be used to oversample T1, T1, and T2 data in the ISIC training dataset. This strategy aims to counteract the underrepresentation of thin lesions, thereby improving the accuracy of deep learning models in identifying early melanoma.

## 5. Conclusions

Deep learning algorithms have been developed to support non-dermatologist clinicians or assist in identifying suspicious lesions prior to clinical care. While significant progress has been made in this field, several key challenges remain unresolved. Our analysis highlights the following issues for deep learning algorithms in detecting malignant melanoma: (1) state-of-the-art algorithms exhibit significantly worse performance on in situ and T1 melanoma images compared to their reported performance on the ISIC Challenge dataset; (2) this results in a substantial drop in performance when evaluated on EMB dataset, which includes mainly early-stage melanoma; and (3) clinical images pose a notable challenge for the tested models, further impacting their effectiveness. A lack of diversity in the training data likely contributes to the described problems. The unknown distribution of melanoma stages and the underrepresentation of early-stage lesions in commonly used datasets introduce biases, leading to early-stage melanoma often being misclassified as benign. Models struggle to effectively transfer their learned features to new data with early-stage cases. This suggests that the visual characteristics of early melanoma are not adequately learned as discriminative features when broader, less-specific datasets are used for training. Misclassification of Tis and T1 category melanomas is a performance deficit that poses a considerable barrier to the clinical applicability of these automated systems, as reliable early detection is critical for improving patient outcomes. Most of the currently developed models use the same data for training and rarely perform a cross-dataset evaluation of model performance. Addressing these challenges is crucial for developing more robust and reliable deep learning systems for melanoma detection. The Early-Stage Melanoma Benchmark dataset provides a valuable resource to help tackle these issues by enabling T-category-specific analysis and cross-dataset evaluation. Through its curated T-category information, it enables testing of model performance on melanomas with varying thicknesses. This ultimately supports the development of automated diagnostic systems that are more robust, clinically relevant, and effective in detecting early-stage melanoma.

## Figures and Tables

**Table 1:** Modified T-category from TNM staging method of melanoma. A and B categories are grouped together [32].

T-Category	Tis	T1	T2	T3	T4
Breslow thickness [mm]	0	≤1.0	>1.0–2.0	>2.0–4.0	>4.0

**Table 2:** Comparison of different models on EMB dataset. Column Rep. shows the average accuracy reported by the authors. Other columns show a fraction of correctly predicted images in the whole dataset and subsets corresponding to the melanoma stage. Models are described by model name, input size, and number of classes the model was trained to recognize. Bold values indicate the T-category where each model achieves its best performance.

Model	Rep.	All	Tis	T1	T2	T3	T4
ConvNeXt-DP 224 9c	0.880	0.445	0.428	0.433	**0.603**	0.471	0.565
ConvNeXt-DMP 224 9c	0.880	0.488	0.455	0.490	0.630	0.588	**0.826**
ConvNeXt-DP 224 2c	0.890	0.414	0.365	0.440	**0.589**	0.588	0.565
ConvNeXt-DMP 224 2c	0.907	0.551	0.532	0.534	**0.740**	0.529	0.739
EfficientNet-B4 448 9c	0.974	0.477	0.431	0.505	0.644	0.529	**0.652**
EfficientNet-B4 896 9c	0.974	0.455	0.418	0.446	0.685	0.588	**0.739**
EfficientNet-B4 640 9c	0.977	0.486	0.469	0.456	**0.699**	0.588	0.696
EfficientNet-B4 768 9c	0.977	0.542	0.519	0.521	0.726	**0.824**	0.696
EfficientNet-B5 640 4c	0.977	0.485	0.463	0.472	**0.658**	0.647	0.609
EfficientNet-B5 640 9c	0.977	0.524	0.494	0.518	**0.740**	0.529	0.739
EfficientNet-B5 448 9c	0.975	0.482	0.461	0.469	0.644	0.588	**0.652**
EfficientNet-B6 448 9c	0.974	0.510	0.488	0.500	0.671	0.588	**0.696**
EfficientNet-B6 576 9c	0.976	0.467	0.446	0.443	0.658	0.647	**0.696**
EfficientNet-B6 640 9c	0.976	0.463	0.421	0.474	**0.685**	0.588	0.565
EfficientNet-B7 576 9c	0.976	0.477	0.456	0.469	0.630	**0.647**	0.565
EfficientNet-B7 640 9c	0.975	0.482	0.455	0.477	0.603	**0.824**	0.652
ResNeSt-101 640 9c	0.973	0.471	0.436	0.469	0.658	0.529	**0.783**
SE-ResNeXt-101 640 9c	0.974	0.501	0.479	0.497	**0.671**	0.529	0.565
ECL 224 9c	0.861	0.395	0.364	0.391	0.548	0.588	**0.652**
ECL 224 8c	0.872	0.214	0.172	0.223	0.397	0.353	**0.478**

## Data Availability

Data and code used in this study are available at ISIC Archive https://gallery.isic-archive.com (accessed on 12 November 2024); Drmoscopy Atlas https://www.dermoscopyatlas.com/ (accessed on 12 November 2024); and GitHub https://github.com/Oichii/EMB (accessed on 20 June 2025).

## Abbreviations

The following abbreviations are used in this manuscript:

ISIC	The International Skin Imaging Collaboration
UMAP	Uniform Manifold Approximation and Projection for Dimension Reduction
EMB	Early Melanoma Benchmark
Grad-CAM	Gradient-weighted Class Activation Mapping
DMP	deep mask pixel-wise supervision
ECL	Class-Enhancement Contrastive Learning for Long-tailed Skin Lesion Classification

## References

[1] Five-Year Survival Rates|SEER Training. https://training.seer.cancer.gov/melanoma/intro/survival.html.

[2] Xu R., Wang C., Zhang J., Xu S., Meng W., Zhang X. (2024). SkinFormer: Learning Statistical Texture Representation with Transformer for Skin Lesion Segmentation. IEEE J. Biomed. Health Inform..

[3] Pandimurugan V., Ahmad S., Prabu A.V., Rahmani M.K.I., Abdeljaber H.A.M., Eswaran M., Nazeer J. (2024). CNN-Based Deep Learning Model for Early Identification and Categorization of Melanoma Skin Cancer Using Medical Imaging. SN Comput. Sci..

[4] Lin T.L., Lu C.T., Karmakar R., Nampalley K., Mukundan A., Hsiao Y.P., Hsieh S.C., Wang H.C. (2024). Assessing the Efficacy of the Spectrum-Aided Vision Enhancer (SAVE) to Detect Acral Lentiginous Melanoma, Melanoma In Situ, Nodular Melanoma, and Superficial Spreading Melanoma. Diagnostics.

[5] Elshahawy M., Elnemr A., Oproescu M., Schiopu A.G., Elgarayhi A., Elmogy M.M., Sallah M. (2023). Early Melanoma Detection Based on a Hybrid YOLOv5 and ResNet Technique. Diagnostics.

[6] Patel R.H., Foltz E.A., Witkowski A., Ludzik J. (2023). Analysis of Artificial Intelligence-Based Approaches Applied to Non-Invasive Imaging for Early Detection of Melanoma: A Systematic Review. Cancers.

[7] Alenezi F., Armghan A., Polat K. (2023). A Novel Multi-Task Learning Network Based on Melanoma Segmentation and Classification with Skin Lesion Images. Diagnostics.

[8] Akash R J N., Kaushik A., Sivaswamy J., Celebi M.E., Salekin M.S., Kim H., Albarqouni S., Barata C., Halpern A., Tschandl P., Combalia M., Liu Y., Zamzmi G. (2023). Evidence-Driven Differential Diagnosis of Malignant Melanoma. Medical Image Computing and Computer Assisted Intervention—MICCAI 2023 Workshops, Proceedings of the MICCAI 2023, Vancouver, BC, Canada, 8–12 October 2023.

[9] Mahmud M.A.A., Afrin S., Mridha M.F., Alfarhood S., Che D., Safran M. (2025). Explainable Deep Learning Approaches for High Precision Early Melanoma Detection Using Dermoscopic Images. Sci. Rep..

[10] Yu Z., Nguyen J., Nguyen T.D., Kelly J., Mclean C., Bonnington P., Zhang L., Mar V., Ge Z. (2022). Early Melanoma Diagnosis with Sequential Dermoscopic Images. IEEE Trans. Med. Imaging.

[11] Polesie S., Gillstedt M., Kittler H., Rinner C., Tschandl P., Paoli J. (2022). Assessment of Melanoma Thickness Based on Dermoscopy Images: An Open, Web-based, International, Diagnostic Study. J. Eur. Acad. Dermatol. Venereol..

[12] Dominguez-Morales J.P., Hernández-Rodríguez J.C., Duran-Lopez L., Conejo-Mir J., Pereyra-Rodriguez J.J. (2025). Melanoma Breslow Thickness Classification Using Ensemble-Based Knowledge Distillation with Semi-Supervised Convolutional Neural Networks. IEEE J. Biomed. Health Inform..

[13] Jaworek-Korjakowska J., Kleczek P., Gorgon M. Melanoma Thickness Prediction Based on Convolutional Neural Network with VGG-19 Model Transfer Learning. Proceedings of the 2019 IEEE/CVF Conference on Computer Vision and Pattern Recognition Workshops (CVPRW).

[14] Nogales M., Acha B., Alarcón F., Pereyra J., Serrano C. (2024). Robust Melanoma Thickness Prediction via Deep Transfer Learning Enhanced by XAI Techniques. arXiv.

[15] Kawahara J., Daneshvar S., Argenziano G., Hamarneh G. (2018). 7-Point Checklist and Skin Lesion Classification Using Multi-Task Multi-Modal Neural Nets. IEEE J. Biomed. Health Inform..

[16] Ferrara G., Argenziano G. (2021). The WHO 2018 Classification of Cutaneous Melanocytic Neoplasms: Suggestions From Routine Practice. Front. Oncol..

[17] Mirikharaji Z., Barata C., Abhishek K., Bissoto A., Avila S., Valle E., Celebi M.E., Hamarneh G. (2022). A Survey on Deep Learning for Skin Lesion Segmentation. arXiv.

[18] Daneshjou R., Smith M.P., Sun M.D., Rotemberg V., Zou J. (2021). Lack of Transparency and Potential Bias in Artificial Intelligence Data Sets and Algorithms: A Scoping Review. JAMA Dermatol..

[19] Hendrycks D., Dietterich T. (2019). Benchmarking Neural Network Robustness to Common Corruptions and Perturbations. arXiv.

[20] Groh M., Harris C., Soenksen L., Lau F., Han R., Kim A., Koochek A., Badri O. (2021). Evaluating Deep Neural Networks Trained on Clinical Images in Dermatology with the Fitzpatrick 17k Dataset. arXiv.

[21] Daneshjou R., Vodrahalli K., Novoa R.A., Jenkins M., Liang W., Rotemberg V., Ko J., Swetter S.M., Bailey E.E., Gevaert O. (2022). Disparities in Dermatology AI Performance on a Diverse, Curated Clinical Image Set. Sci. Adv..

[22] ISIC Archive. https://gallery.isic-archive.com/#!/topWithHeader/onlyHeaderTop/gallery?filter=%5B%5D.

[23] Dermoscopy Atlas|Home. https://www.dermoscopyatlas.com/.

[24] Gutman D., Codella N.C.F., Celebi E., Helba B., Marchetti M., Mishra N., Halpern A. (2016). Skin Lesion Analysis toward Melanoma Detection: A Challenge at the International Symposium on Biomedical Imaging (ISBI) 2016, Hosted by the International Skin Imaging Collaboration (ISIC). arXiv.

[25] Codella N.C.F., Gutman D., Celebi M.E., Helba B., Marchetti M.A., Dusza S.W., Kalloo A., Liopyris K., Mishra N., Kittler H. (2018). Skin Lesion Analysis Toward Melanoma Detection: A Challenge at the 2017 International Symposium on Biomedical Imaging (ISBI), Hosted by the International Skin Imaging Collaboration (ISIC). arXiv.

[26] Codella N., Rotemberg V., Tschandl P., Celebi M.E., Dusza S., Gutman D., Helba B., Kalloo A., Liopyris K., Marchetti M. (2019). Skin Lesion Analysis Toward Melanoma Detection 2018: A Challenge Hosted by the International Skin Imaging Collaboration (ISIC). arXiv.

[27] Combalia M., Codella N.C.F., Rotemberg V., Helba B., Vilaplana V., Reiter O., Carrera C., Barreiro A., Halpern A.C., Puig S. (2019). BCN20000: Dermoscopic Lesions in the Wild. arXiv.

[28] Tschandl P., Rosendahl C., Kittler H. (2018). The HAM10000 Dataset, a Large Collection of Multi-Source Dermatoscopic Images of Common Pigmented Skin Lesions. Sci. Data.

[29] The ISIC 2020 Challenge Dataset. https://challenge2020.isic-archive.com/.

[30] Cassidy B., Kendrick C., Brodzicki A., Jaworek-Korjakowska J., Yap M.H. (2022). Analysis of the ISIC Image Datasets: Usage, Benchmarks and Recommendations. Med. Image Anal..

[31] Mikrut R. (2025). Qarmin/Czkawka. https://github.com/qarmin/czkawka.

[32] Keung E.Z., Gershenwald J.E. (2018). The Eighth Edition American Joint Committee on Cancer (AJCC) Melanoma Staging System: Implications for Melanoma Treatment and Care. Expert Rev. Anticancer. Ther..

[33] Ha Q., Liu B., Liu F. (2020). Identifying Melanoma Images Using EfficientNet Ensemble: Winning Solution to the SIIM-ISIC Melanoma Classification Challenge. arXiv.

[34] Zhang Y., Chen J., Wang K., Xie F. (2023). ECL: Class-Enhancement Contrastive Learning for Long-tailed Skin Lesion Classification. arXiv.

[35] Dzieniszewska A., Garbat P., Piramidowicz R. (2025). Deep Pixel-Wise Supervision for Skin Lesion Classification. Comput. Biol. Med..

[36] Selvaraju R.R., Cogswell M., Das A., Vedantam R., Parikh D., Batra D. (2020). Grad-CAM: Visual Explanations from Deep Networks via Gradient-based Localization. Int. J. Comput. Vis..

